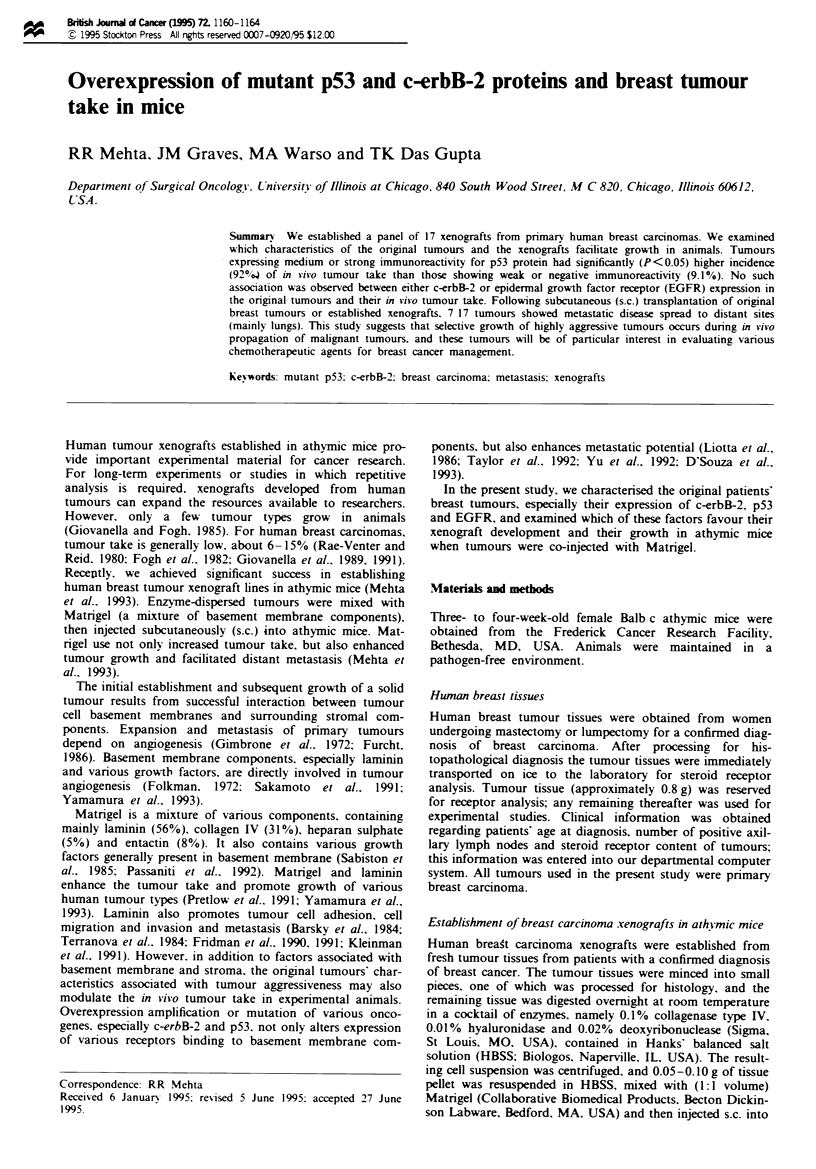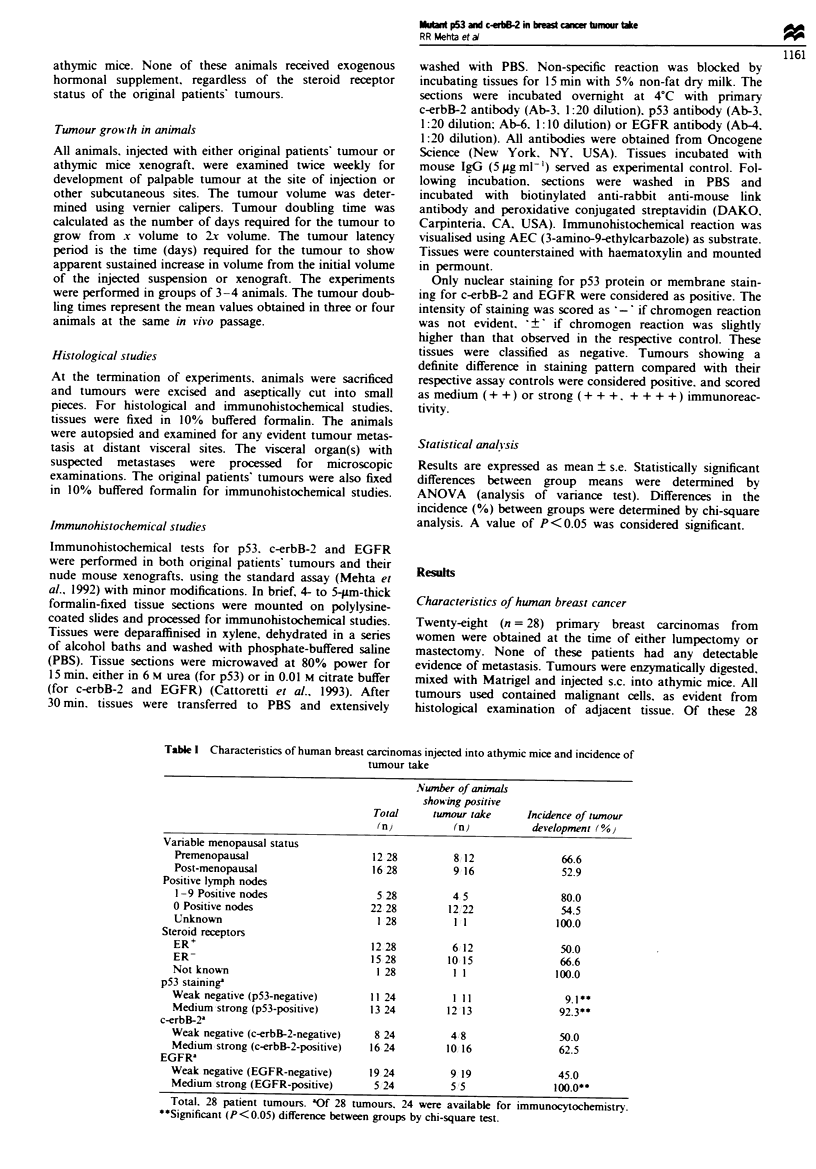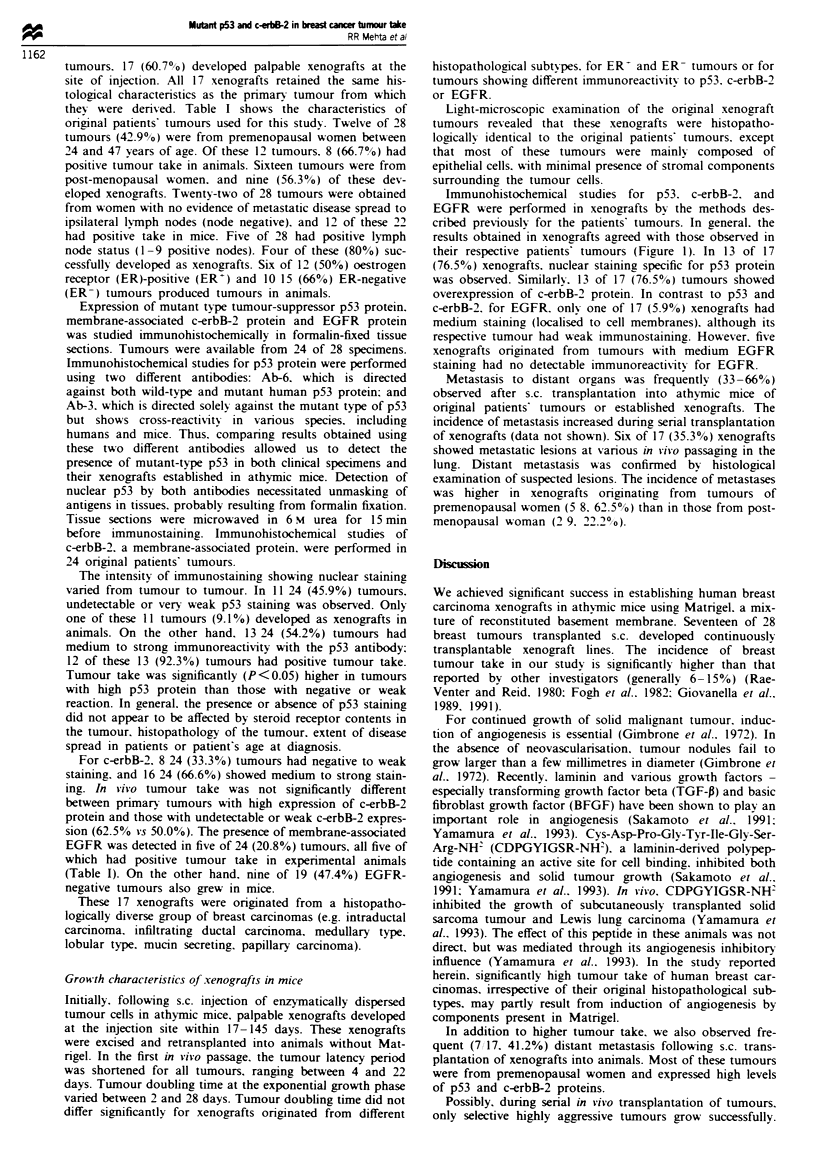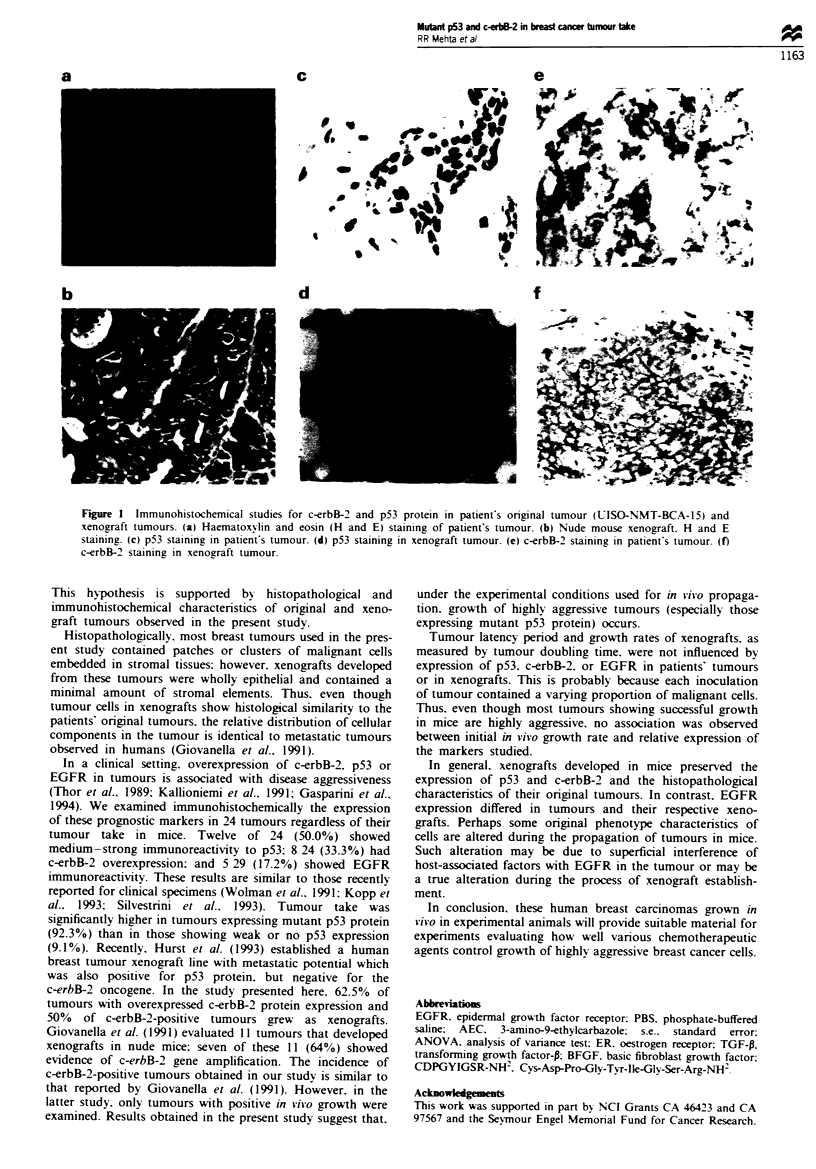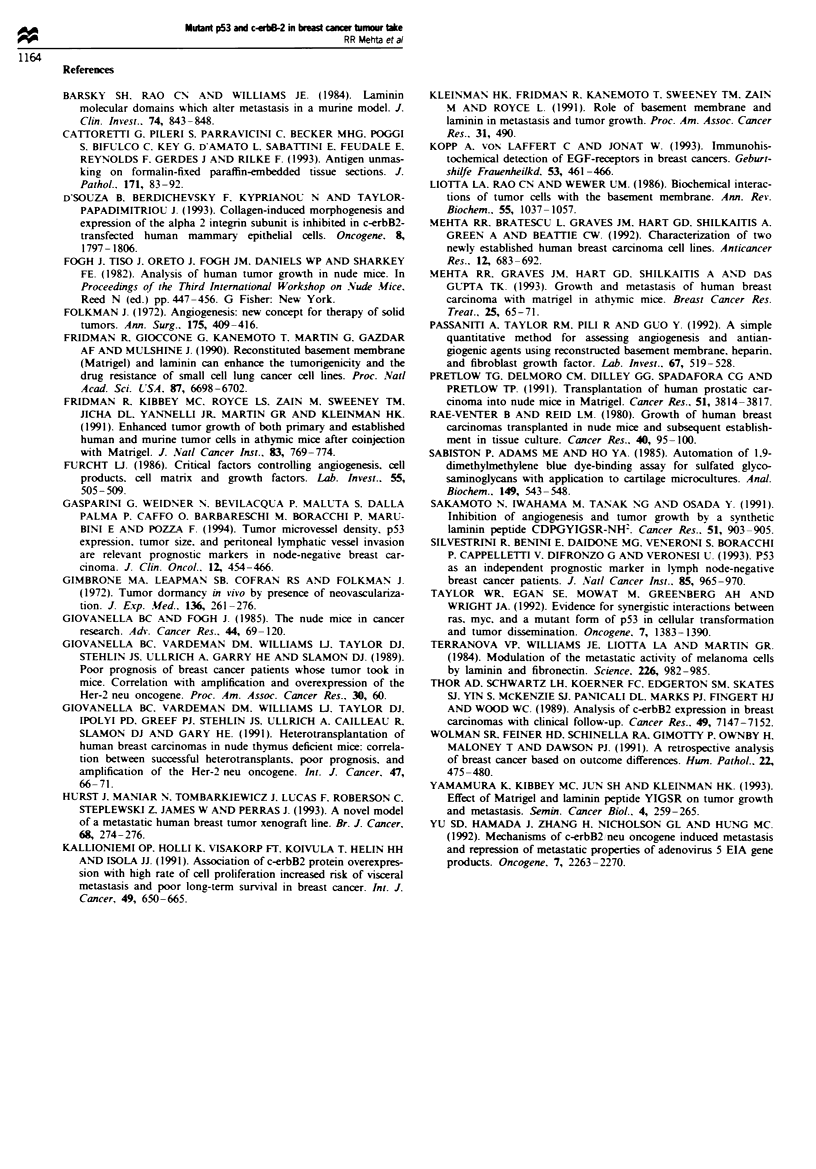# Overexpression of mutant p53 and c-erbB-2 proteins and breast tumour take in mice.

**DOI:** 10.1038/bjc.1995.480

**Published:** 1995-11

**Authors:** R. R. Mehta, J. M. Graves, M. A. Warso, T. K. Das Gupta

**Affiliations:** Department of Surgical Oncology, University of Illinois at Chicago 60612, USA.

## Abstract

**Images:**


British Journal of Cancer (1995) 72, 1160-1164

© 1995 Stockton Press All rights reserved 0007-0920/95 $12.00

### Overexpression of mutant p53 and c-erbB-2 proteins and breast tumour take in mice

RR Mehta, JM Graves, MA Warso and TK Das Gupta

*Department of Surgical Oncology, University of Illinois at Chicago, 840 South Wood Street, M C 820, Chicago, Illinois 60612, USA.*

**Summary** We established a panel of 17 xenografts from primary human breast carcinomas. We examined which characteristics of the original tumours and the xenografts facilitate growth in animals. Tumours expressing medium or strong immunoreactivity for p53 protein had significantly ($P<0.05$) higher incidence (92%) of *in vivo* tumour take than those showing weak or negative immunoreactivity (9.1%). No such association was observed between either c-erbB-2 or epidermal growth factor receptor (EGFR) expression in the original tumours and their *in vivo* tumour take. Following subcutaneous (s.c.) transplantation of original breast tumours or established xenografts, 7/17 tumours showed metastatic disease spread to distant sites (mainly lungs). This study suggests that selective growth of highly aggressive tumours occurs during *in vivo* propagation of malignant tumours, and these tumours will be of particular interest in evaluating various chemotherapeutic agents for breast cancer management.

**Keywords**: mutant p53; c-erbB-2; breast carcinoma; metastasis; xenografts

Human tumour xenografts established in athymic mice provide important experimental material for cancer research. For long-term experiments or studies in which repetitive analysis is required, xenografts developed from human tumours can expand the resources available to researchers. However, only a few tumour types grow in animals (Giovanella and Fogh, 1985). For human breast carcinomas, tumour take is generally low, about 6-15% (Rae-Venter and Reid, 1980; Fogh *et al.*, 1982; Giovanella *et al.*, 1989, 1991). Recently, we achieved significant success in establishing human breast tumour xenograft lines in athymic mice (Mehta *et al.*, 1993). Enzyme-dispersed tumours were mixed with Matrigel (a mixture of basement membrane components), then injected subcutaneously (s.c.) into athymic mice. Matrigel use not only increased tumour take, but also enhanced tumour growth and facilitated distant metastasis (Mehta *et al.*, 1993).

The initial establishment and subsequent growth of a solid tumour results from successful interaction between tumour cell basement membranes and surrounding stromal components. Expansion and metastasis of primary tumours depend on angiogenesis (Gimbrone *et al.*, 1972; Furcht, 1986). Basement membrane components, especially laminin and various growth factors, are directly involved in tumour angiogenesis (Folkman, 1972; Sakamoto *et al.*, 1991; Yamamura *et al.*, 1993).

Matrigel is a mixture of various components, containing mainly laminin (56%), collagen IV (31%), heparan sulphate (5%) and entactin (8%). It also contains various growth factors generally present in basement membrane (Sabiston *et al.*, 1985; Passaniti *et al.*, 1992). Matrigel and laminin enhance the tumour take and promote growth of various human tumour types (Pretlow *et al.*, 1991; Yamamura *et al.*, 1993). Laminin also promotes tumour cell adhesion, cell migration and invasion and metastasis (Barsky *et al.*, 1984; Terranova *et al.*, 1984; Fridman *et al.*, 1990, 1991; Kleinman *et al.*, 1991). However, in addition to factors associated with basement membrane and stroma, the original tumours' characteristics associated with tumour aggressiveness may also modulate the *in vivo* tumour take in experimental animals. Overexpression amplification or mutation of various oncogenes, especially c-*erb*B-2 and p53, not only alters expression of various receptors binding to basement membrane components, but also enhances metastatic potential (Liotta *et al.*, 1986; Taylor *et al.*, 1992; Yu *et al.*, 1992; D'Souza *et al.*, 1993).

In the present study, we characterised the original patients' breast tumours, especially their expression of c-erbB-2, p53 and EGFR, and examined which of these factors favour their xenograft development and their growth in athymic mice when tumours were co-injected with Matrigel.

### Materials and methods

Three- to four-week-old female Balb/c athymic mice were obtained from the Frederick Cancer Research Facility, Bethesda, MD, USA. Animals were maintained in a pathogen-free environment.

### *Human breast tissues*

Human breast tumour tissues were obtained from women undergoing mastectomy or lumpectomy for a confirmed diagnosis of breast carcinoma. After processing for histopathological diagnosis the tumour tissues were immediately transported on ice to the laboratory for steroid receptor analysis. Tumour tissue (approximately 0.8 g) was reserved for receptor analysis; any remaining thereafter was used for experimental studies. Clinical information was obtained regarding patients' age at diagnosis, number of positive axillary lymph nodes and steroid receptor content of tumours; this information was entered into our departmental computer system. All tumours used in the present study were primary breast carcinoma.

### *Establishment of breast carcinoma xenografts in athymic mice*

Human breast carcinoma xenografts were established from fresh tumour tissues from patients with a confirmed diagnosis of breast cancer. The tumour tissues were minced into small pieces, one of which was processed for histology, and the remaining tissue was digested overnight at room temperature in a cocktail of enzymes, namely 0.1% collagenase type IV, 0.01% hyaluronidase and 0.02% deoxyribonuclease (Sigma, St Louis, MO, USA), contained in Hanks' balanced salt solution (HBSS; Biologos, Naperville, IL, USA). The resulting cell suspension was centrifuged, and 0.05-0.10 g of tissue pellet was resuspended in HBSS, mixed with (1:1 volume) Matrigel (Collaborative Biomedical Products, Becton Dickinson Labware, Bedford, MA, USA) and then injected s.c. into

Correspondence: RR Mehta

Received 6 January 1995; revised 5 June 1995; accepted 27 June 1995.

athymic mice. None of these animals received exogenous hormonal supplement, regardless of the steroid receptor status of the original patients' tumours.

*Tumour growth in animals*

All animals, injected with either original patients' tumour or athymic mice xenograft, were examined twice weekly for development of palpable tumour at the site of injection or other subcutaneous sites. The tumour volume was determined using vernier calipers. Tumour doubling time was calculated as the number of days required for the tumour to grow from $x$ volume to $2x$ volume. The tumour latency period is the time (days) required for the tumour to show apparent sustained increase in volume from the initial volume of the injected suspension or xenograft. The experiments were performed in groups of 3–4 animals. The tumour doubling times represent the mean values obtained in three or four animals at the same *in vivo* passage.

*Histological studies*

At the termination of experiments, animals were sacrificed and tumours were excised and aseptically cut into small pieces. For histological and immunohistochemical studies, tissues were fixed in 10% buffered formalin. The animals were autopsied and examined for any evident tumour metastasis at distant visceral sites. The visceral organ(s) with suspected metastases were processed for microscopic examinations. The original patients' tumours were also fixed in 10% buffered formalin for immunohistochemical studies.

*Immunohistochemical studies*

Immunohistochemical tests for p53, c-erbB-2 and EGFR were performed in both original patients' tumours and their nude mouse xenografts, using the standard assay (Mehta *et al.*, 1992) with minor modifications. In brief, 4- to 5-μm-thick formalin-fixed tissue sections were mounted on polylysine-coated slides and processed for immunohistochemical studies. Tissues were deparaffinised in xylene, dehydrated in a series of alcohol baths and washed with phosphate-buffered saline (PBS). Tissue sections were microwaved at 80% power for 15 min, either in 6 M urea (for p53) or in 0.01 M citrate buffer (for c-erbB-2 and EGFR) (Cattoretti *et al.*, 1993). After 30 min, tissues were transferred to PBS and extensively washed with PBS. Non-specific reaction was blocked by incubating tissues for 15 min with 5% non-fat dry milk. The sections were incubated overnight at 4°C with primary c-erbB-2 antibody (Ab-3, 1:20 dilution), p53 antibody (Ab-3, 1:20 dilution; Ab-6, 1:10 dilution) or EGFR antibody (Ab-4, 1:20 dilution). All antibodies were obtained from Oncogene Science (New York, NY, USA). Tissues incubated with mouse IgG ($5\ \mu g\ ml^{-1}$) served as experimental control. Following incubation, sections were washed in PBS and incubated with biotinylated anti-rabbit anti-mouse link antibody and peroxidative conjugated streptavidin (DAKO, Carpinteria, CA, USA). Immunohistochemical reaction was visualised using AEC (3-amino-9-ethylcarbazole) as substrate. Tissues were counterstained with haematoxylin and mounted in permount.

Only nuclear staining for p53 protein or membrane staining for c-erbB-2 and EGFR were considered as positive. The intensity of staining was scored as '−' if chromogen reaction was not evident, '±' if chromogen reaction was slightly higher than that observed in the respective control. These tissues were classified as negative. Tumours showing a definite difference in staining pattern compared with their respective assay controls were considered positive, and scored as medium (+ +) or strong (+ + +, + + + +) immunoreactivity.

*Statistical analysis*

Results are expressed as mean ± s.e. Statistically significant differences between group means were determined by ANOVA (analysis of variance test). Differences in the incidence (%) between groups were determined by chi-square analysis. A value of $P<0.05$ was considered significant.

### Results

*Characteristics of human breast cancer*

Twenty-eight ($n = 28$) primary breast carcinomas from women were obtained at the time of either lumpectomy or mastectomy. None of these patients had any detectable evidence of metastasis. Tumours were enzymatically digested, mixed with Matrigel and injected s.c. into athymic mice. All tumours used contained malignant cells, as evident from histological examination of adjacent tissue. Of these 28

**Table I** Characteristics of human breast carcinomas injected into athymic mice and incidence of tumour take

| | *Total* (n) | *Number of animals showing positive tumour take* (n) | *Incidence of tumour development* (%) |
|---|---|---|---|
| Variable menopausal status | | | |
| Premenopausal | 12/28 | 8/12 | 66.6 |
| Post-menopausal | 16/28 | 9/16 | 52.9 |
| Positive lymph nodes | | | |
| 1–9 Positive nodes | 5/28 | 4/5 | 80.0 |
| 0 Positive nodes | 22/28 | 12/22 | 54.5 |
| Unknown | 1/28 | 1/1 | 100.0 |
| Steroid receptors | | | |
| $ER^+$ | 12/28 | 6/12 | 50.0 |
| $ER^-$ | 15/28 | 10/15 | 66.6 |
| Not known | 1/28 | 1/1 | 100.0 |
| p53 staining[a] | | | |
| Weak/negative (p53-negative) | 11/24 | 1/11 | 9.1** |
| Medium/strong (p53-positive) | 13/24 | 12/13 | 92.3** |
| c-erbB-2[a] | | | |
| Weak/negative (c-erbB-2-negative) | 8/24 | 4/8 | 50.0 |
| Medium/strong (c-erbB-2-positive) | 16/24 | 10/16 | 62.5 |
| EGFR[a] | | | |
| Weak/negative (EGFR-negative) | 19/24 | 9/19 | 45.0 |
| Medium/strong (EGFR-positive) | 5/24 | 5/5 | 100.0** |

Total, 28 patient tumours. [a]Of 28 tumours, 24 were available for immunocytochemistry. **Significant ($P<0.05$) difference between groups by chi-square test.

tumours, 17 (60.7%) developed palpable xenografts at the site of injection. All 17 xenografts retained the same histological characteristics as the primary tumour from which they were derived. Table I shows the characteristics of original patients' tumours used for this study. Twelve of 28 tumours (42.9%) were from premenopausal women between 24 and 47 years of age. Of these 12 tumours, 8 (66.7%) had positive tumour take in animals. Sixteen tumours were from post-menopausal women, and nine (56.3%) of these developed xenografts. Twenty-two of 28 tumours were obtained from women with no evidence of metastatic disease spread to ipsilateral lymph nodes (node negative), and 12 of these 22 had positive take in mice. Five of 28 had positive lymph node status (1–9 positive nodes). Four of these (80%) successfully developed as xenografts. Six of 12 (50%) oestrogen receptor (ER)-positive ($ER^+$) and 10 15 (66%) ER-negative ($ER^-$) tumours produced tumours in animals.

Expression of mutant type tumour-suppressor p53 protein, membrane-associated c-erbB-2 protein and EGFR protein was studied immunohistochemically in formalin-fixed tissue sections. Tumours were available from 24 of 28 specimens. Immunohistochemical studies for p53 protein were performed using two different antibodies: Ab-6, which is directed against both wild-type and mutant human p53 protein; and Ab-3, which is directed solely against the mutant type of p53 but shows cross-reactivity in various species, including humans and mice. Thus, comparing results obtained using these two different antibodies allowed us to detect the presence of mutant-type p53 in both clinical specimens and their xenografts established in athymic mice. Detection of nuclear p53 by both antibodies necessitated unmasking of antigens in tissues, probably resulting from formalin fixation. Tissue sections were microwaved in 6 M urea for 15 min before immunostaining. Immunohistochemical studies of c-erbB-2, a membrane-associated protein, were performed in 24 original patients' tumours.

The intensity of immunostaining showing nuclear staining varied from tumour to tumour. In 11 24 (45.9%) tumours, undetectable or very weak p53 staining was observed. Only one of these 11 tumours (9.1%) developed as xenografts in animals. On the other hand, 13 24 (54.2%) tumours had medium to strong immunoreactivity with the p53 antibody; 12 of these 13 (92.3%) tumours had positive tumour take. Tumour take was significantly ($P<0.05$) higher in tumours with high p53 protein than those with negative or weak reaction. In general, the presence or absence of p53 staining did not appear to be affected by steroid receptor contents in the tumour, histopathology of the tumour, extent of disease spread in patients or patient's age at diagnosis.

For c-erbB-2, 8 24 (33.3%) tumours had negative to weak staining, and 16 24 (66.6%) showed medium to strong staining. *In vivo* tumour take was not significantly different between primary tumours with high expression of c-erbB-2 protein and those with undetectable or weak c-erbB-2 expression (62.5% *vs* 50.0%). The presence of membrane-associated EGFR was detected in five of 24 (20.8%) tumours, all five of which had positive tumour take in experimental animals (Table I). On the other hand, nine of 19 (47.4%) EGFR-negative tumours also grew in mice.

These 17 xenografts were originated from a histopathologically diverse group of breast carcinomas (e.g. intraductal carcinoma, infiltrating ductal carcinoma, medullary type, lobular type, mucin secreting, papillary carcinoma).

*Growth characteristics of xenografts in mice*

Initially, following s.c. injection of enzymatically dispersed tumour cells in athymic mice, palpable xenografts developed at the injection site within 17–145 days. These xenografts were excised and retransplanted into animals without Matrigel. In the first *in vivo* passage, the tumour latency period was shortened for all tumours, ranging between 4 and 22 days. Tumour doubling time at the exponential growth phase varied between 2 and 28 days. Tumour doubling time did not differ significantly for xenografts originated from different histopathological subtypes, for $ER^-$ and $ER^-$ tumours or for tumours showing different immunoreactivity to p53, c-erbB-2 or EGFR.

Light-microscopic examination of the original xenograft tumours revealed that these xenografts were histopathologically identical to the original patients' tumours, except that most of these tumours were mainly composed of epithelial cells, with minimal presence of stromal components surrounding the tumour cells.

Immunohistochemical studies for p53, c-erbB-2, and EGFR were performed in xenografts by the methods described previously for the patients' tumours. In general, the results obtained in xenografts agreed with those observed in their respective patients' tumours (Figure 1). In 13 of 17 (76.5%) xenografts, nuclear staining specific for p53 protein was observed. Similarly, 13 of 17 (76.5%) tumours showed overexpression of c-erbB-2 protein. In contrast to p53 and c-erbB-2, for EGFR, only one of 17 (5.9%) xenografts had medium staining (localised to cell membranes), although its respective tumour had weak immunostaining. However, five xenografts originated from tumours with medium EGFR staining had no detectable immunoreactivity for EGFR.

Metastasis to distant organs was frequently (33–66%) observed after s.c. transplantation into athymic mice of original patients' tumours or established xenografts. The incidence of metastasis increased during serial transplantation of xenografts (data not shown). Six of 17 (35.3%) xenografts showed metastatic lesions at various *in vivo* passaging in the lung. Distant metastasis was confirmed by histological examination of suspected lesions. The incidence of metastases was higher in xenografts originating from tumours of premenopausal women (5 8, 62.5%) than in those from post-menopausal woman (2 9, 22.2%).

### Discussion

We achieved significant success in establishing human breast carcinoma xenografts in athymic mice using Matrigel, a mixture of reconstituted basement membrane. Seventeen of 28 breast tumours transplanted s.c. developed continuously transplantable xenograft lines. The incidence of breast tumour take in our study is significantly higher than that reported by other investigators (generally 6–15%) (Rae-Venter and Reid, 1980; Fogh *et al.*, 1982; Giovanella *et al.*, 1989, 1991).

For continued growth of solid malignant tumour, induction of angiogenesis is essential (Gimbrone *et al.*, 1972). In the absence of neovascularisation, tumour nodules fail to grow larger than a few millimetres in diameter (Gimbrone *et al.*, 1972). Recently, laminin and various growth factors – especially transforming growth factor beta (TGF-$\beta$) and basic fibroblast growth factor (BFGF) have been shown to play an important role in angiogenesis (Sakamoto *et al.*, 1991; Yamamura *et al.*, 1993). Cys-Asp-Pro-Gly-Tyr-Ile-Gly-Ser-Arg-$NH^2$ (CDPGYIGSR-$NH^2$), a laminin-derived polypeptide containing an active site for cell binding, inhibited both angiogenesis and solid tumour growth (Sakamoto *et al.*, 1991; Yamamura *et al.*, 1993). *In vivo*, CDPGYIGSR-$NH^2$ inhibited the growth of subcutaneously transplanted solid sarcoma tumour and Lewis lung carcinoma (Yamamura *et al.*, 1993). The effect of this peptide in these animals was not direct, but was mediated through its angiogenesis inhibitory influence (Yamamura *et al.*, 1993). In the study reported herein, significantly high tumour take of human breast carcinomas, irrespective of their original histopathological subtypes, may partly result from induction of angiogenesis by components present in Matrigel.

In addition to higher tumour take, we also observed frequent (7/17, 41.2%) distant metastasis following s.c. transplantation of xenografts into animals. Most of these tumours were from premenopausal women and expressed high levels of p53 and c-erbB-2 proteins.

Possibly, during serial *in vivo* transplantation of tumours, only selective highly aggressive tumours grow successfully.

**Figure 1** Immunohistochemical studies for c-erbB-2 and p53 protein in patient's original tumour (UISO-NMT-BCA-15) and xenograft tumours. (**a**) Haematoxylin and eosin (H and E) staining of patient's tumour. (**b**) Nude mouse xenograft, H and E staining. (**c**) p53 staining in patient's tumour. (**d**) p53 staining in xenograft tumour. (**e**) c-erbB-2 staining in patient's tumour. (**f**) c-erbB-2 staining in xenograft tumour.

This hypothesis is supported by histopathological and immunohistochemical characteristics of original and xenograft tumours observed in the present study.

Histopathologically, most breast tumours used in the present study contained patches or clusters of malignant cells embedded in stromal tissues; however, xenografts developed from these tumours were wholly epithelial and contained a minimal amount of stromal elements. Thus, even though tumour cells in xenografts show histological similarity to the patients' original tumours, the relative distribution of cellular components in the tumour is identical to metastatic tumours observed in humans (Giovanella *et al*., 1991).

In a clinical setting, overexpression of c-erbB-2, p53 or EGFR in tumours is associated with disease aggressiveness (Thor *et al*., 1989; Kallioniemi *et al*., 1991; Gasparini *et al*., 1994). We examined immunohistochemically the expression of these prognostic markers in 24 tumours regardless of their tumour take in mice. Twelve of 24 (50.0%) showed medium–strong immunoreactivity to p53; 8 24 (33.3%) had c-erbB-2 overexpression; and 5 29 (17.2%) showed EGFR immunoreactivity. These results are similar to those recently reported for clinical specimens (Wolman *et al*., 1991; Kopp *et al*., 1993; Silvestrini *et al*., 1993). Tumour take was significantly higher in tumours expressing mutant p53 protein (92.3%) than in those showing weak or no p53 expression (9.1%). Recently, Hurst *et al*. (1993) established a human breast tumour xenograft line with metastatic potential which was also positive for p53 protein, but negative for the c-*erb*B-2 oncogene. In the study presented here, 62.5% of tumours with overexpressed c-erbB-2 protein expression and 50% of c-erbB-2-positive tumours grew as xenografts. Giovanella *et al*. (1991) evaluated 11 tumours that developed xenografts in nude mice; seven of these 11 (64%) showed evidence of c-*erb*B-2 gene amplification. The incidence of c-erbB-2-positive tumours obtained in our study is similar to that reported by Giovanella *et al*. (1991). However, in the latter study, only tumours with positive *in vivo* growth were examined. Results obtained in the present study suggest that, under the experimental conditions used for *in vivo* propagation, growth of highly aggressive tumours (especially those expressing mutant p53 protein) occurs.

Tumour latency period and growth rates of xenografts, as measured by tumour doubling time, were not influenced by expression of p53, c-erbB-2, or EGFR in patients' tumours or in xenografts. This is probably because each inoculation of tumour contained a varying proportion of malignant cells. Thus, even though most tumours showing successful growth in mice are highly aggressive, no association was observed between initial *in vivo* growth rate and relative expression of the markers studied.

In general, xenografts developed in mice preserved the expression of p53 and c-erbB-2 and the histopathological characteristics of their original tumours. In contrast, EGFR expression differed in tumours and their respective xenografts. Perhaps some original phenotype characteristics of cells are altered during the propagation of tumours in mice. Such alteration may be due to superficial interference of host-associated factors with EGFR in the tumour or may be a true alteration during the process of xenograft establishment.

In conclusion, these human breast carcinomas grown *in vivo* in experimental animals will provide suitable material for experiments evaluating how well various chemotherapeutic agents control growth of highly aggressive breast cancer cells.

**Abbreviations**

EGFR, epidermal growth factor receptor; PBS, phosphate-buffered saline; AEC, 3-amino-9-ethylcarbazole; s.e., standard error; ANOVA, analysis of variance test; ER, oestrogen receptor; TGF-$\beta$, transforming growth factor-$\beta$; BFGF, basic fibroblast growth factor; CDPGYIGSR-$NH^2$, Cys-Asp-Pro-Gly-Tyr-Ile-Gly-Ser-Arg-$NH^2$.

**Acknowledgements**

This work was supported in part by NCI Grants CA 46423 and CA 97567 and the Seymour Engel Memorial Fund for Cancer Research.

**References**

BARSKY SH. RAO CN AND WILLIAMS JE. (1984). Laminin molecular domains which alter metastasis in a murine model. *J. Clin. Invest.*, **74**, 843-848.

CATTORETTI G. PILERI S. PARRAVICINI C. BECKER MHG. POGGI S. BIFULCO C. KEY G. D'AMATO L. SABATTINI E. FEUDALE E. REYNOLDS F. GERDES J AND RILKE F. (1993). Antigen unmasking on formalin-fixed paraffin-embedded tissue sections. *J. Pathol.*, **171**, 83-92.

D'SOUZA B. BERDICHEVSKY F. KYPRIANOU N AND TAYLOR-PAPADIMITRIOU J. (1993). Collagen-induced morphogenesis and expression of the alpha 2 integrin subunit is inhibited in c-erbB2-transfected human mammary epithelial cells. *Oncogene*, **8**, 1797-1806.

FOGH J. TISO J. ORETO J. FOGH JM. DANIELS WP AND SHARKEY FE. (1982). Analysis of human tumor growth in nude mice. In *Proceedings of the Third International Workshop on Nude Mice*. Reed N (ed.) pp. 447-456. G Fisher: New York.

FOLKMAN J. (1972). Angiogenesis: new concept for therapy of solid tumors. *Ann. Surg.*, **175**, 409-416.

FRIDMAN R. GIOCCONE G. KANEMOTO T. MARTIN G. GAZDAR AF AND MULSHINE J. (1990). Reconstituted basement membrane (Matrigel) and laminin can enhance the tumorigenicity and the drug resistance of small cell lung cancer cell lines. *Proc. Natl Acad. Sci. USA*, **87**, 6698-6702.

FRIDMAN R. KIBBEY MC. ROYCE LS. ZAIN M. SWEENEY TM. JICHA DL. YANNELLI JR. MARTIN GR AND KLEINMAN HK. (1991). Enhanced tumor growth of both primary and established human and murine tumor cells in athymic mice after coinjection with Matrigel. *J. Natl Cancer Inst.*, **83**, 769-774.

FURCHT LJ. (1986). Critical factors controlling angiogenesis, cell products, cell matrix and growth factors. *Lab. Invest.*, **55**, 505-509.

GASPARINI G. WEIDNER N. BEVILACQUA P. MALUTA S. DALLA PALMA P. CAFFO O. BARBARESCHI M. BORACCHI P. MARUBINI E AND POZZA F. (1994). Tumor microvessel density, p53 expression, tumor size, and peritoneal lymphatic vessel invasion are relevant prognostic markers in node-negative breast carcinoma. *J. Clin. Oncol.*, **12**, 454-466.

GIMBRONE MA. LEAPMAN SB. COFRAN RS AND FOLKMAN J. (1972). Tumor dormancy *in vivo* by presence of neovascularization. *J. Exp. Med.*, **136**, 261-276.

GIOVANELLA BC AND FOGH J. (1985). The nude mice in cancer research. *Adv. Cancer Res.*, **44**, 69-120.

GIOVANELLA BC. VARDEMAN DM. WILLIAMS LJ. TAYLOR DJ. STEHLIN JS. ULLRICH A. GARRY HE AND SLAMON DJ. (1989). Poor prognosis of breast cancer patients whose tumor took in mice. Correlation with amplification and overexpression of the Her-2 neu oncogene. *Proc. Am. Assoc. Cancer Res.*, **30**, 60.

GIOVANELLA BC. VARDEMAN DM. WILLIAMS LJ. TAYLOR DJ. IPOLYI PD. GREEF PJ. STEHLIN JS. ULLRICH A. CAILLEAU R. SLAMON DJ AND GARY HE. (1991). Heterotransplantation of human breast carcinomas in nude thymus deficient mice: correlation between successful heterotransplants, poor prognosis, and amplification of the Her-2 neu oncogene. *Int. J. Cancer*, **47**, 66-71.

HURST J. MANIAR N. TOMBARKIEWICZ J. LUCAS F. ROBERSON C. STEPLEWSKI Z. JAMES W AND PERRAS J. (1993). A novel model of a metastatic human breast tumor xenograft line. *Br. J. Cancer*, **68**, 274-276.

KALLIONIEMI OP. HOLLI K. VISAKORP FT. KOIVULA T. HELIN HH AND ISOLA JJ. (1991). Association of c-erbB2 protein overexpression with high rate of cell proliferation increased risk of visceral metastasis and poor long-term survival in breast cancer. *Int. J. Cancer*, **49**, 650-665.

KLEINMAN HK. FRIDMAN R. KANEMOTO T. SWEENEY TM. ZAIN M AND ROYCE L. (1991). Role of basement membrane and laminin in metastasis and tumor growth. *Proc. Am. Assoc. Cancer Res.*, **31**, 490.

KOPP A. VON LAFFERT C AND JONAT W. (1993). Immunohistochemical detection of EGF-receptors in breast cancers. *Geburtshilfe Frauenheilkd.* **53**, 461-466.

LIOTTA LA. RAO CN AND WEWER UM. (1986). Biochemical interactions of tumor cells with the basement membrane. *Ann. Rev. Biochem.*, **55**, 1037-1057.

MEHTA RR. BRATESCU L. GRAVES JM. HART GD. SHILKAITIS A. GREEN A AND BEATTIE CW. (1992). Characterization of two newly established human breast carcinoma cell lines. *Anticancer Res.*, **12**, 683-692.

MEHTA RR. GRAVES JM. HART GD. SHILKAITIS A AND DAS GUPTA TK. (1993). Growth and metastasis of human breast carcinoma with matrigel in athymic mice. *Breast Cancer Res. Treat.*, **25**, 65-71.

PASSANITI A. TAYLOR RM. PILI R AND GUO Y. (1992). A simple quantitative method for assessing angiogenesis and antiangiogenic agents using reconstructed basement membrane, heparin, and fibroblast growth factor. *Lab. Invest.*, **67**, 519-528.

PRETLOW TG. DELMORO CM. DILLEY GG. SPADAFORA CG AND PRETLOW TP. (1991). Transplantation of human prostatic carcinoma into nude mice in Matrigel. *Cancer Res.*, **51**, 3814-3817.

RAE-VENTER B AND REID LM. (1980). Growth of human breast carcinomas transplanted in nude mice and subsequent establishment in tissue culture. *Cancer Res.*, **40**, 95-100.

SABISTON P. ADAMS ME AND HO YA. (1985). Automation of 1,9-dimethylmethylene blue dye-binding assay for sulfated glycosaminoglycans with application to cartilage microcultures. *Anal. Biochem.*, **149**, 543-548.

SAKAMOTO N. IWAHAMA M. TANAK NG AND OSADA Y. (1991). Inhibition of angiogenesis and tumor growth by a synthetic laminin peptide CDPGYIGSR-$NH^2$. *Cancer Res.*, **51**, 903-905.

SILVESTRINI R. BENINI E. DAIDONE MG. VENERONI S. BORACCHI P. CAPPELLETTI V. DIFRONZO G AND VERONESI U. (1993). P53 as an independent prognostic marker in lymph node-negative breast cancer patients. *J. Natl Cancer Inst.*, **85**, 965-970.

TAYLOR WR. EGAN SE. MOWAT M. GREENBERG AH AND WRIGHT JA. (1992). Evidence for synergistic interactions between ras, myc, and a mutant form of p53 in cellular transformation and tumor dissemination. *Oncogene*, **7**, 1383-1390.

TERRANOVA VP. WILLIAMS JE. LIOTTA LA AND MARTIN GR. (1984). Modulation of the metastatic activity of melanoma cells by laminin and fibronectin. *Science*, **226**, 982-985.

THOR AD. SCHWARTZ LH. KOERNER FC. EDGERTON SM. SKATES SJ. YIN S. MCKENZIE SJ. PANICALI DL. MARKS PJ. FINGERT HJ AND WOOD WC. (1989). Analysis of c-erbB2 expression in breast carcinomas with clinical follow-up. *Cancer Res.*, **49**, 7147-7152.

WOLMAN SR. FEINER HD. SCHINELLA RA. GIMOTTY P. OWNBY H. MALONEY T AND DAWSON PJ. (1991). A retrospective analysis of breast cancer based on outcome differences. *Hum. Pathol.*, **22**, 475-480.

YAMAMURA K. KIBBEY MC. JUN SH AND KLEINMAN HK. (1993). Effect of Matrigel and laminin peptide YIGSR on tumor growth and metastasis. *Semin. Cancer Biol.*, **4**, 259-265.

YU SD. HAMADA J. ZHANG H. NICHOLSON GL AND HUNG MC. (1992). Mechanisms of c-erbB2 neu oncogene induced metastasis and repression of metastatic properties of adenovirus 5 EIA gene products. *Oncogene*, **7**, 2263-2270.